# Radionuclides Landscape in Prostate Cancer Theranostics

**DOI:** 10.3390/ijms26146751

**Published:** 2025-07-14

**Authors:** Monica Neagu, Carolina Constantin, Mihail Eugen Hinescu, Petrisor Gabriel Bleotu, Mara-Georgiana Popovici, Maria-Iulia Zai, Klaus Michael Spohr

**Affiliations:** 1 “Victor Babes” National Institute, 99-01 Splaiul Independentei, 050096 Bucharest, Romania; caroconstantin@gmail.com (C.C.); mhinescu@yahoo.com (M.E.H.); 2 Colentina Clinical Hospital, 19-21 Soseaua Stefan cel Mare, 020125 Bucharest, Romania; 3 Faculty of Biology, University of Bucharest, 91-95 Splaiul Independentei, 050107 Bucharest, Romania; 4 Department of Cellular and Molecular Biology and Histology, “Carol Davila” University of Medicine and Pharmacy, 8 Bulevardul Eroii Sanitari, 050474 Bucharest, Romania; 5 Extreme Light Infrastructure-Nuclear Physics (ELI-NP), “Horia Hulubei” National Institute for Physics and Nuclear Engineering (IFIN-HH), 30 Reactorului Street, 077125 Magurele, Romania; gabriel.bleotu@eli-np.ro (P.G.B.); mara.popovici@eli-np.ro (M.-G.P.); iulia.zai@eli-np.ro (M.-I.Z.); klaus.spohr@eli-np.ro (K.M.S.); 6 Doctoral School of Applied Science, National University of Science and Technology Politehnica of Bucharest, Splaiul Independentei 313, 060042 Bucharest, Romania; 7 School of Computing, Engineering and Physical Sciences, University of the West of Scotland, High Street, Paisley PA1 2BE, UK

**Keywords:** prostate cancer, radiotherapy, theranostic, radionuclides, prostate-specific membrane antigen

## Abstract

Prostate cancer, a malignancy of significant prevalence, affects approximately half a million men in Europe, with one in twelve males receiving a diagnosis before reaching the age of 75. Radiotheranostics represents a paradigm shift in prostate cancer treatment, leveraging radionuclides for diagnostic and therapeutic applications, with PSMA emerging as the primary molecular target. Regulatory bodies have approved various PSMA-targeted radiodiagnostic agents, such as [^18^F]DCFPyL (PYLARIFY^®^, Lantheus Holdings), [^18^F]rhPSMA-7.3 (POSLUMA^®^, Blue Earth Diagnostics), and [^68^Ga]Ga-PSMA-11 (LOCAMETZ^®^, Novartis/ILLUCCIX^®^, Telix Pharmaceuticals), as well as therapeutic agents like [^177^Lu]Lu-PSMA-617 (PLUVICTO^®^, 15 Novartis). The approval of PLUVICTO^®^ in March 2022 for patients with metastatic castration-resistant prostate cancer who have undergone prior treatments, including androgen receptor pathway-targeting agents and taxane-based chemotherapy, represents a significant advancement. Other radionuclides like ^161^Tb, ^149^Tb, ^225^Ac, ^227^Th, ^223^Ra, ^211^At, ^213^ Bi, ^212^Pb, ^89^Zr, and ^125^I are presented, emphasizing their clinical implementation or the stage of clinical trial they are in in the flow to biomedical implementation. Three clinically wise used radionuclides ^177^Lu, ^225^Ac, ^223^Ra are shown along with their characteristics. This review aims to elucidate the molecular mechanisms underpinning PSMA, explore the clinical applications of PSMA-targeted radiotheranostics, and critically examine the diverse challenges these therapies encounter in the treatment of prostate cancer.

## 1. Introduction

Excluding non-melanoma skin cancers, prostate cancer is the most prevalent malignancy among men in Europe, following breast, colorectal, and lung cancers, affecting approximately half a million individuals. One in twelve men will receive a diagnosis of prostate cancer before the age of seventy-five, with a mortality risk of 1 in 103 in Europe [1]. The introduction of the Prostate-Specific Antigen (PSA) test in the 1990s markedly increased early-stage diagnoses, leading to a corresponding rise in incidence rates [2]. By 2020, approximately 1.4 million new cases of prostate cancer were documented globally. Localized prostate cancer would imply monitoring strategy, radical surgery, radiation therapy (brachytherapy or external beam), and emergent focal therapies. Localized prostate cancer has a heterogeneous natural history and can evolve from indolent to aggressive tumors with metastatic variants, which would imply additional treatments. Clinical management typically targets the androgen receptor (AR) pathway through interventions such as androgen deprivation, anti-androgens, and neoadjuvant chemotherapy. However, resistance to therapy generally develops within 6–12 months, resulting in progression to castration-resistant prostate cancer (CRPC) [3].

Prostate cancers (PCs) exhibit considerable heterogeneity, generally associated with the serum biomarker PSA. Within this category, CRPC can include asymptomatic patients with elevated PSA following hormone blockade failure, as well as those with rapidly progressive disease leading to mortality. The progression of metastatic disease remains a focus of intensive research. Patient characteristics, including younger age, high Gleason score (>7) [4], elevated PSA, rapid PSA doubling time, and increased serum alkaline phosphatase, may predispose individuals to metastasis [5]. Despite geographical disparities in access to modern treatments [6], the therapeutic landscape for metastatic prostate cancer has improved significantly over the past 15 years. Androgen deprivation therapy (ADT) has been the standard care for metastatic prostate cancer, yet survival outcomes have been limited, prompting the development of novel treatment options [7]. Due to the limited survival outcomes under this therapy, many new treatment options have been developed in the last few years. Regarding the hormone-sensitive prostate cancer, combination therapies of two or three agents of ADT, AR signaling inhibitors (ARSI), and chemotherapy have been established and further led to a significant improvement in overall survival.

In metastatic patients, effective therapies include docetaxel and cabazitaxel chemotherapy, abiraterone and enzalutamide (ARSI), olaparib and rucaparib (Poly-ADP-ribose Polymerase Inhibitors—PARPi), sipuleucel-T as immunotherapy, and two radiopharmaceutical agents (radium-223 and lutetium-177 ([177Lu]Lu-PSMA-617), with these compounds culminating in the recent FDA approvals of gallium-68-based [68Ga]Ga-PSMA-11 and [18F]F-DCFPyL, which have enhanced the scope of diagnostic and therapeutic radioligands for radionuclide theranostics. These radionuclides are utilized for positron emission tomography (PET) imaging to detect metastases and facilitate PSMA-targeted therapy [8].

Radiotheranostics has rapidly advanced across various solid tumors, with [177Lu]Lu-PSMA-617 approved for prostate cancer treatment and commercially designated Pluvicto^®^. The therapy targets PSMA on the surface of tumor cells and is employed alongside [68Ga] gozetotide (LOCAMETZ^®^, NOVARTIS, Basel, Switerland) as an imaging agent [9,10,11]. Radiotheranostics, initially employed in cancer management, emerged from nuclear medicine, demonstrating the considerable potential for future advancements pertaining to both radioisotopes and molecular targets [12].

In the VISION clinical trial, patients treated with [177Lu]Lu-PSMA-617 exhibited notable clinical benefits compared to standard care alone; however, therapy remains challenging, as only approximately 50% of patients demonstrate a positive clinical response. Predicting absorbed dose may enhance patient selection, and a 2022 study utilized [68Ga]Ga-PSMA-PET scans to forecast absorbed doses of [^177^Lu]Lu-PSMA-617, facilitating personalized treatment [13].

During the year 2022, guidelines recommended 68Ga-PSMA-11 PET-CT following local therapy to detect residual active tumors. Challenges persist concerning PSMA accumulation in non-target tissues, including the vesicoureteral junction. Furthermore, ADT may lead to misinterpretation of tracer uptake, as long-term ADT reduces PSMA expression and tracer uptake. At the same time, short-duration ADT can enhance PSMA expression, resulting in further misinterpretations [14]. A 2023 study involving over 130 patients indicated that [18F]F-PSMA-1007-PET/CT could effectively identify local relapses [15].

Upon diagnosis of high-risk localized disease, multimodal treatment strategies are essential. Various radiation modalities and combinations are employed in managing high-risk prostate cancer; however, clinical outcomes remain suboptimal. Therefore, personalizin treatment based on cellular and molecular tumor characteristics as well as patient-specific factors is an essential future objective in prostate cancer management [16,17].

This review will focus on the radiotherapeutic approaches to prostate cancer and the associated challenges of metastatic disease. Without being exhaustive, the review will tackle prostatic-specific antigen and its specific targeted radiotherapy, with the focus on ^177^Lu as the main theranostic radionuclide. Besides ^177^Lu, other radionuclides are presented along with the limitations of PSMA-based therapies. Moreover, our paper emphasizes theranostics featuring various radionuclides besides ^177^Lu and highlight the molecular mechanisms of PSMA-targeted therapy. Interesting facts are presented regarding therapeutical insights into PC as a result of endeavors synergizing RT and other approved therapies in oncology. Thus, our paper gathers updated information on various radionuclides in PC before critically evaluating them in terms of their physics and, of course, their clinical efficiency. The review ends with data on the last ongoing radionuclide clinical studies in the domain, specifically aligning with ^177^Lu clinical trials entering the approval stage. Last but not least, a few indicators for the recent advancements in PSMA-targeted therapeutics in prostate cancers trying to overcome the limitations of the PSMA-based therapies are also presented in our work.

## 2. Prostate-Specific Membrane Antigen

**Figure 1:**
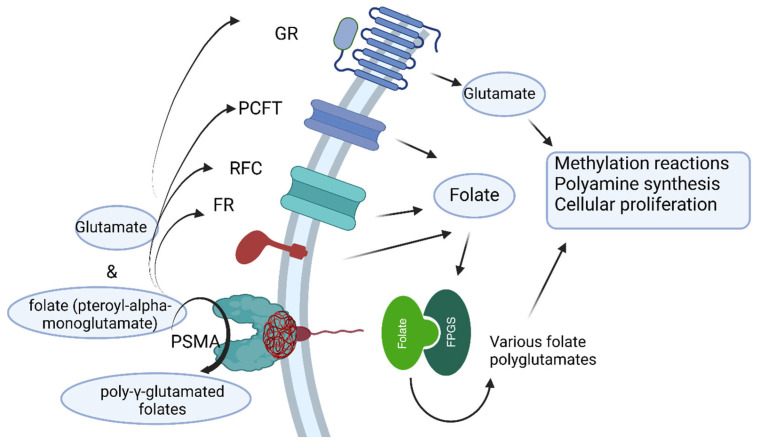
Enzymatic activity pathways of PSMA loop involved in prostate tumorigenesis. PSMA transforms poly-γ-glutamated folates secreted by dead tumor cells to folate and glutamate. Folate is then taken up by living tumor cells through the proton-coupled folate transporter (PCFT), folate receptor (FR), and reduced folate carrier (RFC). Glutamate is taken up by the glutamate receptor (GR). Within the cell, folate is polyglutamated in the polyamine and nucleotide synthesis cycles as well as the methylation reactions for cell proliferation. GR after taking up the glutamate can stimulate methylation reactions, polyamine synthesis, and cellular proliferation yet again. Created in Biorender. Monica Neagu (2025).

PSMA, is a transmembrane glycoprotein expressed by, but not restricted to, the epithelial cells of the prostate. In high-grade prostate cancer, PSMA is translocated to the luminal surface of the ducts and is overexpressed. Therefore, it is a molecule highly expressed on the membrane of most prostate cancer cells and its expression is correlated with Gleason score and castrate resistance status. PSMA is expressed to a lesser extent in normal tissues (prostate, small intestine, salivary glands (SGs) and lachrymal glands, kidneys, and even in other malignancy like renal cell carcinoma). When a ligand binds to the PSMA, it is internalized in the cell. This property provides an interesting target for both diagnosis and therapy [18]. PSMA’s involvement in metabolic pathways like the glutamate and folate potentially confers a survival advantage under conditions of cellular stress [19]. Seminal studies have elucidated that PC is characterized by molecular hallmarks, including aberrant signaling of AR and Phosphoinositide 3-kinases (PI3K) pathways [20,21], in addition to PSMA overexpression. PSMA has been demonstrated to activate PI3K–Akt signaling by liberating glutamate as a messenger molecule [22]. Furthermore, PSMA regulation is predicted on intricate interactions with the AR, involving PI3K/Akt signaling and in DNA damage repair (DDR) mechanisms. Advancements in our understanding of PSMA biology will undoubtedly inform the development of biomarkers to pin-point resistance mechanisms to PSMA-based therapies, thereby improving outcomes for PC patients [23]. PSMA exhibits dual catalytic activities: N-acetylated alpha-linked acidic dipeptidase (NAALDase) and folate hydrolase. Both enzymatic functions result in the liberation of glutamate, with the folate hydrolase activity of PSMA being a subject of intense investigation in PC [24]. A schematic representation of PSMA’s enzymatic activities, cycle, and involvement in prostate tumorigenesis is presented in Figure 1.

Although localized PCs are primarily managed through surgical intervention or radiotherapy or a combination of both, advanced hormone-refractory metastatic disease, specifically metastatic castration-resistant prostate cancer (mCRPC), remains characterized by substantial morbidity and mortality rates [1].

PSMA has emerged as a pivotal target in the realm of prostate cancer research and treatment. A plethora of PSMA inhibitors, predicated on phosphorous-, thiol-, and urea-based molecules, as well as macrocyclic chelators, have been meticulously developed. PSMA, a transmembrane glycoprotein, exhibits markedly elevated expression levels concomitant with tumor invasiveness [25].

In recent years, theranostic approaches have utilized a diminutive PSMA inhibitor molecule (1,4,7,10-tetraazacyclododecane-1,4,7,10-tetraacetic acid-DOTA), conjugated with 177Lu, culminating in the compound designated as Lu-PSMA-617. This innovative agent is both an imaging probe and a radioactive therapeutic modality in mCRPC [26]. Contemporary research endeavors are focused on enhancing drug efficacy through augmenting specific affinity and mitigating off-target toxicity. To this end, chemical modifications of the linkage between targeting structures and imaging payloads are being assiduously investigated, heralding the advent of next-generation therapeutic agents [8]. Notwithstanding multiple PSMA-targeted treatments, the optimal selection of patients and prognostication of therapeutic resistance remain formidable clinical challenges. Elevated PSMA expression is associated with disease progression and perturbations of the DDR mechanisms [23].

**Figure 2:**
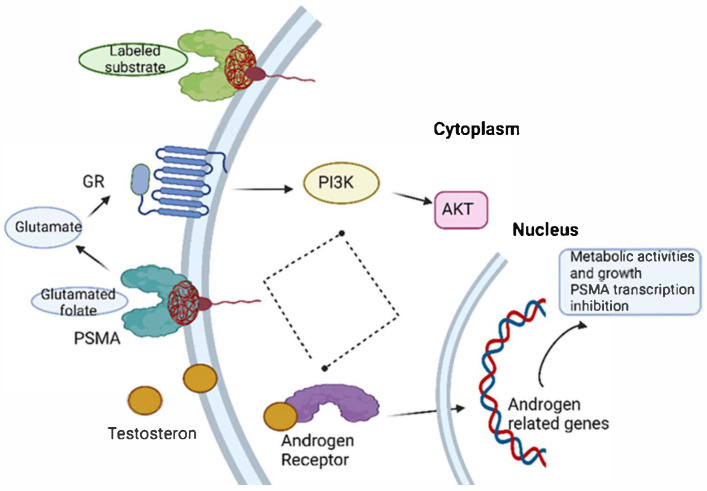
Therapy targets involving PSMA and intracellular networks triggered by PSMA. In PC, glutamate is a signaling molecule linking membrane glutamate receptor (GR) to the intracellular activation of the PI3K-AKT pathway. The androgen receptor axis interplays with the GR through the PI3K-AKT pathway; thus, it can be a regulatory loop that therapy approaches can trigger (depicted with a dashed line). Androgen receptor activation can induce the transcription of androgen-related genes that regulate metabolic activities, as well as PSMA transcription inhibition. [^177^Lu]Lu-PSMA-617 (depicted as labeled substrate for PSMA) is developed for theranostic approaches. Created in Biorender. Monica Neagu (2025).

In addition to PSMA’s significance as a therapeutic target, the AR pathway remains the principal driver of prostate cancer progression, as its activation augments the transcription of genes regulating cellular proliferation and metabolism. In CRPC, inhibitors of AR signaling (e.g., enzalutamide, abiraterone acetate) have become the standard of care, although therapeutic resistance inevitably emerges in a subset of patients. Multiple molecular pathways are implicated in the genesis of CRPC; one such pathway is the dysregulated PI3K pathway, which is ubiquitously observed in CRPC patients. A reciprocal feedback loop exists between AR and PI3K signaling pathways, potentially interpreting the mechanisms of resistance acquisition in CRPC [27]. Recent investigations have implicated PSMA as a PI3K–AKT–mTOR signaling modulator. Specifically, PSMA’s enzymatic activity induces AKT signaling and subsequent glutamate release. These findings demonstrate a direct relationship between AR and PSMA. Moreover, a combinatorial therapeutic approach utilizing PI3K and AR inhibitors may offer enhanced clinical outcomes. Figure 2 delineates the principal therapeutic intervention sites, with PSMA as the central molecular entity. This molecule exhibits low expression on normal cells, high expression on cancer cells, and is localized on the cell surface. Consequently, it represents an ideal candidate for direct radiolabeling or targeting through specific pharmaceutical inhibitors [28]. PSMA has been implicated in therapy resistance and in the progression of PC to an untreatable stage [22].

## 3. PSMA-Targeted Endoradiotherapy

PSMA-targeted therapies employ radiopharmaceuticals that specifically bind to PSMA, facilitating the direct delivery of radiation to cancer cells [29]. This targeted approach minimizes damage to surrounding healthy tissues while enhancing treatment efficacy. To achieve this, the molecular alterations in neoplastically transformed tissue increase the expression of numerous proteins or receptors, all of which can be exploited as therapeutic targets. The portfolio of medical isotopes emits various types of ionizing radiation, most prominently alpha (α) and beta (β) particles, as well as gamma (γ) rays, each possessing distinct therapeutic applications [30].

Alpha (4He)-emitting ions, such as actinium-225 (225Ac) and lead-212 (212Pb) [31], exhibit high energy and short penetration depths due to the high linear energy transfer (LET) of the emitted *α*-particles. The high LET allows for localized destruction of tumors via double-strand DNA breaks while sparing adjacent healthy tissue [32]. The range of *α*-radiation is typically limited to a few micrometers, approximately the size of a few malignant cells, making it ideally suited for targeted therapy.

Beta emitters diversify in two modalities: β^−^nuclei, suchas 177Lu, in which an electron is emitted; and β^+^-isotopes such as ^68^Ga, in which the electron’s antiparticle, a positron, is emitted. For the latter, it is worth noting that the annihilation of the positron results in two energetic γ-photons of 511 keV, which is the core of PET spectroscopy. Beta emitters are utilized in targeted radiotherapy to provide a broader treatment effect suitable for localized and metastatic diseases. The radiation effects can extend from several millimeters to approximately 1 cm around the source’s position. The expelled electron in the β^−^ process also loses energy via LET, albeit 100 to 1000 times lower per unit length compared to α−radiation. Consequently, only single-strand DNA breaks are induced in the cells [33].

The LET process for electrons and positrons is almost equal in magnitude. Like 177Lu, beta-emitting radionuclides often release small amounts of γ-radiation, which generally has a lower LET value compared to beta radiation and disperses its energy more evenly along its path. Additionally, lutetium, the β^+^-emitter 68Ga, is a diagnostic/imaging radionuclide. Copper-based agents, such as 61Cu and 67Cu, extend the arsenal for imaging and therapy with ligands like sarcophagi cage amine [34].

Gamma emitters, such as 99mTc, are primarily employed in diagnostic imaging but can also assist in therapeutic planning for prostate cancer. The LET value for γ-radiation is typically less than 0.3 keV/µm in water; thus, this type of radiation is not spatially confined to the point of emission, penetrating through the entire body, analogous to the use of an external X-ray source [35].

Additionally, Auger electron emission is utilized in targeted therapies to enhance the destruction of cancer cells while minimizing damage to surrounding healthy tissue. Auger electrons are emitted by radioisotopes such as 125I (125-iodine) in the aftermath of the radioactive isotope’s transmutation, e.g., internal conversion process by electron capture (EC). Due to the low energy of Auger electrons, a relatively high LET is prevalent, resulting in short ranges between a few nanometers up to about 100 μm and, hence, very localized damage. This targeted approach allows for more effective treatment of tumors, particularly in cases where conventional therapies may harm adjacent normal tissues [36].

As of 2025, only a few agents have been evaluated in clinical trials, of which none have yet been approved for routine treatment. In March 2025 FDA expanded the approval of ^177^Lu vipivotide tetraxetan to patients with PSMA-positive mCRPC previously treated with the androgen receptor pathway inhibitor [37].

### 3.1. Lutetium-177

Theranostics, a paradigm employing a specific ligand for both diagnostic and therapeutic purposes, was initially conceived with radiotherapies in mind [38]. The aforementioned overexpression of PSMA on prostate cancer cells has been developed for both imaging and targeted radionuclide therapy. Beta- and alpha-emitting particles are employed alongside PSMA as ligands. Adverse effects may arise from targeted radionuclide therapies, including xerostomia and myelosuppression [39]. An evaluation of 250 studies involving 1200 patients diagnosed with mCRPC revealed that over 50% exhibited a reduction in PSA levels with [^177^Lu]Lu-PSMA-617 treatment, accompanied by low toxicity [40]. ^177^Lu is a radioisotope that allows for precise measurement, enabling the calculation of radiation doses and thereby ensuring treatment safety and efficacy. However, further research is necessary to enhance clinical outcomes for patients [41]. Approved in 2022 for mCRPC, [^177^Lu]Lu-PSMA-617 has demonstrated favorable clinical responses in retrospective studies, with re-challenge using [^177^Lu]Lu-PSMA-617 therapy shown to be both safe and effective. Nonetheless, not all patients respond to this therapy, primarily due to the heterogeneity of PSMA expression in prostate tissue that has undergone a neoplastic transformation. PSMA is encoded by the FOLH1 (folate hydrolase 1) gene, governed by a complex regulatory genetic network that may exhibit individual variations. Inter-tumoral and inter-patient heterogeneity in PSMA expression, molecular dynamics, and the tumor microenvironment can significantly impact PSMA-based theranostics [42]. A combination of low-dose Taxol-based chemotherapy with [^177^Lu]Lu-PSMA-617 therapy overcame tumor resistance in mCRPC. In this case, re-challenge with [^177^Lu]Lu-PSMA-617 was combined with a low-dose chemotherapy agent used as a radiosensitizer. Tumor resistance was not registered in this combined therapy case, and patient and scan evaluation showed a remarkable improvement in number, size, and intensity of lesions [43].

A retrospective study published in 2024, involving over 200 patients diagnosed with mCRPC and treated with ^177^Lu PSMA across various European centers, concluded on the efficacy and potential biomarkers for monitoring patient outcomes. After treatment cycles, a PSA decrease of over 30% was observed in nearly 80% of patients following the third treatment cycle. Evaluation of gamma-glutamyl transferase at baseline levels exceeding 30 U/L was correlated with a 1.5-fold-higher risk of progression for non-metastatic patients [44]. Another biomarker assessed in PC patients and reported in 2024 indicated that tumor volume is a prognostic indicator. PSMA-PET/CT facilitates the evaluation of tumor volume, and the PROMISE prostate cancer registry demonstrates that the location of PC metastases prognosticates tumor aggressiveness. A limitation of this method is that decreasing PSMA expression is not necessarily linked to a positive therapeutic response [45]. Utilizing [^177^Lu]Lu-PSMA-617 as radiopharmaceutical continues to present various clinical challenges, particularly regarding optimal combinatorial regimens with other therapies and the formulation of personalized treatment strategies incorporating prognostic biomarkers [46]. Recent developments concerning [^177^Lu]Lu-PSMA-617 therapies, including mechanisms of action, safety, and efficacy, are comprehensively detailed by Ritawidya et al. [26]. Patients treated with PLUVICTO^®^ may experience significant side effects in the hours following a therapeutic dose of 7.4 MBq of ^177^Lu-vipivotide tetraxetan; thus, early discharge should be approached with caution owing to persistent radioactivity in circulation [47]. A study published in 2024 across multiple cancer centers concerning mCRPC treated with [^177^Lu]Lu-PSMA-617 radioligand reported favorable clinical responses in over 160 patients, with more than 50% demonstrating a significant PSA response; side effects included anemia and thrombocytopenia [48].

^177^Lu, recently tested in combination with other isotopes, is another endeavor to improve efficacy. An approach reported in 2021 showed the results of ^177^Lu testing in mCRPC patients in combination with ^225^Ac-PSMA. The enrolled patients were exhibiting visceral metastases, high tumor burden, and bone metastases with a PSA doubling time of under two months. The therapy was tested as monotherapy or as a combination, and a good clinical outcome was registered. Partial remission was observed in over 5% of patients, with progression-free survival (PFS) and overall survival (OS) durations recorded at 9.1 and 14.8 months, respectively, without serious acute adverse effects. The results are auspicious for this clinically challenging cohort [49].

### 3.2. Other Isotopes Used in Targeted Radionuclid Therapy of Prostate Cancer

Integrating ^68^Ga-labeled, PSMA-based PET imaging with radionuclide therapy has been tested in patients with mCRPC, demonstrating both efficacy and tolerability. This radiotheranostic approach facilitates the detection of PC lesions and their molecular characteristics via PET imaging, thereby enabling the personalization of treatment, monitoring of clinical responses, and prediction of patient outcomes [50]. Terbium-161 (^161^Tb)-PSMA-based therapies were investigated for radiotheranostics in mCRPC in 2023, with no treatment-related adverse events reported in ongoing clinical trials, the results of which are eagerly anticipated [51].

^225^Ac has been tested as a PSMA-targeted therapeutic agent, which also targets hK2 (a serine protease expressed by the prostate 80% similar in sequence with PSA) and CD46 (a protein found overexpressed in prostate cancer tissues). However, technological challenges in the production of ^225^Ac necessitate the exploration of other alpha-emitting isotopes (e.g., ^227^Th (thorium-227), ^223^Ra (radium-223), ^211^At (astatine-211) [52], ^213^Bi (bismuth-213), ^212^Pb (lead-212), or ^149^Tb (terbium-149)) [53]. Ongoing clinical trials investigating ^225^Ac-PSMA in mCRPC aim to enhance hormonal therapies, chemotherapy, PARP inhibitors, and immunotherapy to personalize treatment strategies [54].

Stereotactic body radiotherapy (SBRT) in patients with intermediate- to high-risk PC was evaluated using multiparametric MRI and ^68^Ga-PSMA-11 imaging, with recurrence rates documented in 39.4% of patients. Post-therapy recurrences predominantly occurred at the original site or near the primary tumor [55]. Additionally, ^18^F-fluorodeoxyglucose ([^18^F]F-FDG) was assessed for monitoring PSMA through PET/CT imaging, with an emphasis on determining prognostic dynamic parameters. Non-responders to [^177^Lu]Lu-PSMA-617 were evaluated using imaging modalities, including [^18^F]F-FDG and [^68^Ga]-PSMA-11. Potential biomarkers such as glucometabolic activity (cGA) and the one associated with PSMA expression (cGAP) were explored in this study, with cGAP emerging as a predictor of overall survival in this challenging patient’s cohort [56].

In late-stage mCRPC patients, the therapeutic efficacy and associated toxicities of [^177^Lu]Lu-PSMA-617 and ^225^Ac-PSMA-617 were compared. Toxicities affecting the SGs were assessed in terms of SG volume, revealing a statistically significant reduction in volume within the ^225^Ac-PSMA-617 cohort [57]. The approvals of [^177^Lu]Lu-PSMA-617, ^68^Ga-PSMA-11, and [^18^F]F-DCFPyL have paved the way for the exploration of ^225^Ac-PSMA within the theranostic domain. Despite the commendable clinical efficacy of [^177^Lu]Lu-PSMA-617, approximately 30% of patients lack response to this treatment. Conversely, ^225^Ac demonstrates compatibility with favorable clinical outcomes, albeit with the caveat of emitting four highly hazardous, high-energy particles and possessing a prolonged half-life of 10 days [58]. To mitigate potential damage to healthy tissues, encapsulation of radionuclides within nano-carriers has been proposed. This strategy may enhance the absorption of radionuclides by tumor cells while concurrently reducing adverse effects on surrounding healthy tissues [59].

Using ^18^F-fluciclovine for PET/CT imaging has been investigated to assess clinical response to docetaxel in mCRPC. The correlation between PET imaging responses and PSA levels was observed in nearly 43% of patients after one cycle of chemotherapy and in 75% after six cycles, indicating that ^18^F-fluciclovine imaging correlates more effectively with PSA responses compared to conventional CT in mCRPC [60]. Biochemical recurrence (BR), defined as the elevation of PSA levels following surgical or radiotherapeutic interventions, was evaluated in a prospective multicenter study conducted in 2022. This study, involving 17 centers across 15 countries and over 1000 patients, utilized ^68^Ga-PSMA-11 imaging. Notably, over 65% of patients exhibited positive PSMA PET/CT results, which correlated with Gleason scores and PSA levels, thereby demonstrating the utility of PSMA PET/CT in predicting BR in PC patients [61].

Fluorocholine PET was employed to image oligorecurrent pelvic nodal relapses in PC across 15 centers. The findings indicated that the combination of high-dose salvage pelvic radionuclide therapy and ADT significantly prolonged tumor control, with nearly 50% of patients achieving complete remission after three years [62]. In cases where patients progressed under [^177^Lu]Lu-PSMA-617, the alternative ^225^Ac-PSMA may be considered. Lunger et al. reported that xerostomia was the predominant side effect, leading to therapy discontinuation in approximately 25% of patients receiving ^225^Ac-PSMA [63].

With a longer half-life of *t*_1/2_ = 78.4 h, ^89^Zr (89-zirconium) linked to PSMA has been evaluated for PET imaging, allowing for scans to be conducted even six days post-injection. In patients with undetectable PSMA-positive tumors via ^68^Ga-PSMA-11 or ^18^F-JK-PSMA-617, PET scans utilizing ^89^Zr-PSMA-DFO were performed. This study, published in 2022, demonstrated that ^89^Zr-PSMA successfully identified tumors in nearly 60% of patients, manifesting as localized recurrences, lymph node metastases, or distant metastases. The findings suggest that patients exhibiting weak PSMA expression may require an extended duration for ligand internalization, rendering lesions undetectable on PET/CT scans with approved radioligands. Consequently, ^89^Zr-PSMA may provide a solution to this challenge and could be pivotal in the management of weak PSMA-positive tumors [64]. Iodine-125 is a significant radioisotope mainly used as a standalone treatment modality, brachytherapy, for localized prostate cancer. This low-dose-rate (LDR) therapy involves the direct implantation of radioactive seeds into the prostate, allowing for a concentrated radiation dose to the tumor while minimizing exposure to adjacent tissues [65,66]. Various clinical studies have demonstrated the efficacy of ^125^I brachytherapy, showing improved cure rates and disease-free survival [67]. The decay process of ^125^I is primarily through electron capture (EC), with a relatively long half-life of *t*_1/2_ = 59.4 d suited for brachytherapy. A substantial percentage of patients achieve a return to baseline in PSA levels post-treatment, indicating effective disease control [68]. Additionally, the procedure is associated with manageable side effects, further contributing to its acceptance as a viable treatment option [69].

Additionally, ^213^Bi binding to the PSMA-617 antigen was investigated for the treatment of mCRPC-PSMA-targeted alpha therapy [70] and its use was found particularly appealing due to its ability to deliver high-energy radiation over short distances via α-particle and β-particle decay, which can effectively kill cancer cells while minimizing collateral damage to surrounding healthy tissue [71].

Table 1A provides a comprehensive overview of the most significant theranostic radionuclides, referred to as ’Nuclide’ used in prostate cancer therapy. It includes each nuclide’s primary radioactive decay pathway, its half-life, and the average range (r_avg_) of the emitted particles’ trajectory in human tissue. For β^+^-emitters, this range indicates the spatial resolution achievable with PET as the pair of 511 keV-photons emerging from the annihilation radiation originates at the endpoint of the positrons path. The “Purpose” column outlines the primary medical application of each nuclide and the average radioactive activity, A, delivered to a patient in a single treatment session. A complete treatment usually consists of 2 to 8 treatment cycles. Wherever only preliminary data for the animal model is available, a placeholder (“-“) is used. The status of the nuclide approval for theranostic treatment is indicated and if FDA approval has been obtained, the year of the first approval of the associated therapeutic ’Brand/Agent’, is specified. If no therapeutic brand has been released yet (indicated by a (“-“), this column will list the most researched agent related to the treatment. If more than one ’Agent’ has been developed the ’Status’ column diversifies into two sub-rows. The ’Number of patients’ column offers a rough estimation of the number of patients who have been treated with the aforementioned brands and/or have been imagistically investigated for diagnostics. The table shows the tremendously reduced dose rate for α-treatment with ^223^Ra and ^225^Ac. Additionally, the potential for ultra-precisely localized Auger electron-based treatment for ^134^Ce can be deduced from its very low value for ravg. As the research undertaken is on a mixture of both ^134^Ce and ^134^La isotopes, the table will summarize the data in one row. Table 1B details some of the clinical trials that are mentioned in Table 1A.

Several radiotheranostic agents based on PSMA have been approved, including [^18^F]DCFPyL (PYLARIFY^®^, Lantheus Holdings, Bedford, MA 01730, USA), [^18^F]rhPSMA-7.3 (POSLUMA^®^, Blue Earth Diagnostics, Oxford, UK), [^68^Ga]Ga-PSMA-11 (LOCAMETZ^®^, Novartis/ILLUCCIX^®^, Telix Pharmaceuticals, Melbourne, Australia), and [^177^Lu]Lu-PSMA-617 (PLUVICTO^®^, Novartis, Basel, Switzerland). Despite the recent development of the radiotheranostic arsenal, challenges persist in clinical practice [72] as detailed below. Radiation resistance presents a challenge in PC treatment. It is influenced at the molecular level by factors such as cell cycle regulation, DNA damage repair, hypoxic conditions, oxidative stress, testosterone levels, epithelial–mesenchymal transition, and tumor stem cells, just to mention a few mechanisms [73].

### 3.3. PSMA-Based Therapy Limitations

There are several limitations of PSMA-based therapies. Thus, PSMA expression is not confined to prostatic tissue; it was also identified in SGs, kidneys, gastrointestinal mucosa, and even in other types of malignancies [74]. As mentioned above, xerostomia is a severe side effect of PSMA-targeted therapy, due to the SGs’ uptake of PSMA ligands and is currently dose-limiting for ^225^Ac-based therapies [75]. In the kidney’s proximal tubules, PSMA expression was also found [76], linking it to the significant renal toxicity of the therapy, making kidneys one of the most important dose-limiting organs for PSMA-related therapies [77].

PSMA expression in the jejunal brush border of the intestine explains the low-grade gastrointestinal toxicities mainly in ^225^Ac-based radionuclide therapy [78]. Interestingly, PSMA was also found to be expressed in the glial cells of the central nervous system, and in a recent case series, it was shown that brain metastases of mCRPC patients could have a good clinical outcome upon [^177^Lu]Lu-PSMA-617 treatment [79]. Patient variability regarding PSMA expression is another important limitation factor. For example, the loss of PSMA expression on cancer cells was shown in two clinical trials, TheraP and VISION. These clinical trials have emphasized a more stringent criteria for patient selection on the basis of PSMA expression that would undoubtedly increase therapeutic efficacy [10,80]. Heterogeneity in PSMA expression was depicted not only between patients, but within the same tumor, definitely affecting the clinical outcomes [81]. PSMA expression at diagnosis can vary when compared to the progression of the disease; therefore, tumors do not progress mainly in high levels of PSMA expression [82]. Moreover, intratumoral PSMA-high and PSMA-negative cell populations can coexist [81]. Overcoming intratumoral heterogeneity of PSMA expression can be accomplished using various mechanisms. For example, in antibody–drug conjugates (ADCs), lipophilic payloads can circulate extracellularly from PSMA-positive cancer cells to the negative ones and kill them using this beneficial bystander effect [83].

There are various pathways followed by recent studies aiming to overcome the limitations of PSMA-based therapies. Designing the next generation of therapeutic radionuclides, e.g., ^161^Tb-, ^225^Ac, and ^212^Pb, to aid or to replace ^177^Lu. These new radionuclides can have improved efficacy and reduced toxicity. Theranostic radionuclides are designed to optimize imaging and therapeutic dosimetry, taking into account specificity, biodistribution, and metabolism. In this sense, radio hybrid systems can be obtained by chemically bonding distinct radionuclide pairs [83]. Comprehensive additional information on the limitations of PSMA-based therapies could be found in several studies [81,82,84].

**Table 1:** (**A**) Overview of the most prominent radionuclides used or under investigation for prostate cancer treatment as of 2025. (**B**) Details of clinical trials presented in (**A**).

(A)											
Nuclide	Decay	t1/2	ravg	Purpose	A	Status	Year	Brand Name/Agent		Nb. P	Ref.
^18^F	β^+^	109.8 min	2 mm	Imaging	350 MBq	FDA	2021	PYLARIFY/F-18 piflufolastat		≈400–500 k	[8]
							2023	POSLUMA/F-18 flotufolastat		≈5–15 k	[8]
^61^Cu	β^+^	3.33 h	2 mm	Imaging	-	Phase I/II		Cu-61-NuriPro		≈50–150	[85]
^64^Cu	β^+^	12.7 h	6 mm	Imaging	350 MBq	Phase III		(-)/Cu-64-SAR-bisPSMA		≈500–1000	[31]
^67^Cu	β^−^	2.58 d	2 mm	Therapy	12 GBq	Phase I/II		(-)/Cu67-SAR-bisPSMA		≈50–150	[86]
^68^Ga	β^+^	68 min	8 mm	Imaging	300 MBq	FDA	2020	LOCAMETZ/Ga-68 gozetotide		≈10–30 k	[8,9]
							2021	ILLUCCIX/Ga-68 gozetotide		≈50–100 k	
^89^Zr	β^+^	78.4 h	2 mm	Imaging	-	Phase I/II		(-)/Zr-89-DFO-huJ591		≈200–500	[64]
^125^I	EC	59.39 d	<10 µm	Theranostic	15 kBq	FDA	1986	Brachytherapy/No Agent		≈10–20 k	[68]
^161^Tb	β^−^	6.89 d	30 µm	Theranostic	6.5 GBq	Phase I/II		Tb-161-PSMA-I&T		≈50–150 k	[87,88]
^177^Lu	β^−^	6.65 d	230 µm	Theranostic	7.4 GBq	FDA	2022	PLUVICTO/Lu-177 vipivotide		≈15–30 k	[1,9,10]
							2024	(TBA)/177Lu PSMA-I&T		≈1–5 k	[89]
^211^At	α	7.2 h	70 µm	Theranostic	72 MBq	Phase I		(-)/At-211-PSMA-5		≈20–50	[52,90]
^212^Pb	β^−^	10.6 h	100 µm	Theranostic	60 MBq	Phase I/II		(-)/Pb-212-PSMA		<100	[31,91]
^213^Bi	α, *β*^−^	46.6 min	80 µm	Therapy	300 MBq	Preclinical		(-)/Bi-213-DOTATOC 7		-	[70,71,92]
^223^Ra	α	11.4 d	70 µm	Therapy	4.4 MBq	FDA	2013	XOFIGO/Ra-223 dichloride		≈50–100 k	[93]
^225^Ac	α	9.91 d	100 µm	Therapy	9.5 MBq	Phase I/II		(-)/Ac-225-PSMA-617		≈700–900	[49,53]
^134^Ce	Auger	3.16 d	500 nm	Imaging	-	Preclinical		(-)/(-)		-	[93,94]
^134^La	β^+^	6.45 min	1 mm								
**(B)**											
**Radionuclide**		**Company**	**Purpose**	**Phase**	**Evaluation**		**Start** **Date**	**Advantages/Disadvantages**	**Reference**		
^61^Cu-NuriPro^TM^		Nuclidium	Imaging	Non-randomized phase 1 trial	Safety and efficacy when compared to an ^18^F-based, FDA-approved PSMA-targeting radiotracer		2024	Advantages: 3.3-hour half-life, greater distribution post-production, easy manufacture, delayed imaging, detection of small metastases.	Public Releases—Nuclidium (https://nuclidium.com/category/public-releases, accessed on 14 June 2025)		
^61^Cu-NODAGA-PSMA I&T			Therapy	Phase I	To be announced		2025	To be announced			
^64^Cu-SAR-bisPSMA		Clarity	Imaging	Phase III non-randomized, single-arm, open-label, multicenter trial	Imaging efficiency for 220 patients with BR; detection of metastases; efficacy will be assessed on both same-day imaging (Day 1, day of administration) and next-day imaging (Day 2, approximately 24 h post-administration)		2025	Advantages: fast track designation for PET imaging of prostate-specific membrane, antigen-positive, prostate cancer lesions with suspected metastasis in patients who are candidates for initial definitive therapy.	^64^Cu-SAR-bisPSMA in Prostate Cancer and Prostate Cancer Recurrent and Cryotherapy—Clinical Trials Registry—ICH GCP (https://ichgcp.net/clinical-trials-registry/NCT06970847, accessed on 14 June 2025)		
		COBRA		Phase I/II	52 patients with BR and negative or equivocal standard of care imaging; detection of metastases; efficacy will be assessed on Day 0 and Day 1 (1–4h and 24 ± 6h post-dose)		2024	Safe and effective in detecting BR PC patients; in patients with a negative or equivocal SOC scan, it identified lesions in up to 80% of patients and more upon next-day imaging.	Clinical trial information: NCT05249127 (https://www.clinicaltrials.gov/study/NCT05249127, accessed on 14 June 2025)		
^89^Zr-DFO-huJ591			imaging	Phase I/II	50 patients with progressive mCRPC; comparison to whole-body PET/CT scans assessment with bone scintigraphy and cross-sectional imaging		2015	Superior targeting of bone lesions relative to CT; soft-tissue lesions imaging less optimal to CT	[95]		
^161^Tb--PSMA-I&T		VIOLET	Imaging/ therapy	Single-center, single-arm, phase I/II trial	30 patients with progressive, metastatic, castration-resistant prostate cancer		2024	Safety dose; dose escalation at three time-point intervals (2–6 h, 18–24 h, and 72–120 h) after the first cycle of ^161^Tb-PSMA-I&T.	NCT05521412 (https://clinicaltrials.gov/study/NCT05521412/, accessed on 14 June 2025)		
^211^At-PSMA-5			Imaging/ therapy	First-in-human SPECT/CT image of [^211^At]PSMA-5	15 PC patients		2024	Shortest half-life (7.2 h); high therapeutic effect, low abscopal effect, no patient isolation; produced domestically using cyclotron; low renal accumulation at 24h.	SNMMI 2025: First Data of a Novel PSMA-Targeting Radiopharmaceutical Compound SMS-5368: Exploratory and Phase 1/2 Results (https://www.urotoday.com/conference-highlights/snmmi-2025/161575-snmmi-2025-first-data-of-a-novel-psma-targeting-radiopharmaceutical-compound-sms-5368-exploratory-and-phase-1-2-results.html, accessed on 14 June 2025)		
^212^Pb-PSMA		[^212^Pb]Pb-ADVC001	Imaging	Prospective, open-label, non-randomized, dose escalation and optimization	Imaging three patients with progressive mCRPC and ECOG 1; PSA levels 0.44, 0.75, 15 µg/L, 3 metastatic lesions		2024	Safely administered to mCRPC patients; γ-camera imaging; metastatic targeting, with good biodistribution and clearance.	NCT05720130 (https://www.clinicaltrials.gov/study/NCT05720130, accessed on 14 June 2025)		
^225^Ac--PSMA-617		Novartis	Therapy	Phase I, open-label dose escalation study for safety	99 patients enrolled up to 2027 with PSMA-positive prostate cancer who have and have not had prior exposure to [177Lu]Lu-PSMA-617 (177Lu-PSMA-617) or [177Lu]Lu-PSMA I&T (177Lu-PSMA I&T)		2025	Study participation of each participant is approximately 18–24 months (12 months from enrollment to end of each treatment (EOT) plus 12 months of long-term follow-up).	NCT04597411 (https://clinicaltrials.gov/study/NCT04597411, accessed on 14 June 2025)		

Nb. P—Number of Patients treated and/or investigated through imaging.

## 4. Clinical Trials

One of the formidable challenges encountered in high-risk PC surgery is the intraoperative delineation of positive resection margins (PRMs). Recent studies have explored the efficacy of radionuclide techniques in this clinical domain; specifically, the Cerenkov luminescence imaging (CLI) employing ^68^Ga-PSMA-11.

The PRMs approach was tested in seven high-risk PC patients who underwent radical prostatectomy following preoperative [^18^F]-PSMA PET/CT for clinical validation. The study concluded that [18F]-PSMA flexible autoradiography (FAR) CLI exhibited superior tracer-related signal detectability compared to CLI [96].

Further investigations are underway concerning the theranostic potential of specific radionuclides, such as copper-64 (^64^Cu)-DOTHA2-PSMA for PET imaging in PC [97].

The first-in-human imaging with ^61^Cu successfully detected distant metastases in prostate cancer patients [85].

In the radiopharmaceutical therapy domain, abundant preclinical studies have led to the clinical approval of PSMA targeting with β-particle-emitting ^177^Lu in patients with mCRPC who have failed prior chemotherapy [16]. Compared with standard of care, [^177^Lu]Lu-PSMA-617 had low toxicity profiles leading to the actual approval [98]. While it is an important therapeutic step, limitations arise in the clinical setting and hence clinical data suggest further improvement. Thus, preclinical research focuses now on improving [^177^Lu]Lu-PSMA-617 efficacy and developing other PSMA-based radiopharmaceuticals. The combination of linkers and various chelating agents are sought after to increase high and prolonged tumor uptake of the radionuclide through PSMA. Extensive studies on structure–activity relationship focusing on various classes of diagnostic and therapeutic radioligands for radionuclide theranostics are ongoing [8,99]. β-emitting alternatives to ^177^Lu such as ^67^Cu, ^161^Tb, and many more are tested in preclinical settings (see Table 1A).

α-particle-based diagnostic and therapeutic radioligands for radionuclide theranostics were extensively tested in preclinical studies to aid β-particle-based therapeutics. ^223^RaCl_2_ is an α-particle-emitting agent (see Table 1A), which was the first FDA-approved α-particle-emitting agent. It demonstrated important clinical benefits in mCRPC patients displaying bone metastasis. ^223^RaCl_2_ has a high LET, compared to β-particle-emitting counterparts like ^88^Sr and ^153^Sm. Similarly, impressive treatment responses have been achieved using an α-particle-emitting PSMA-based agent, ^225^Ac-PSMA-617, in patients with mCRPC [100,101].

## 5. Recent Advancements in PSMA-Targeted Endotherapeutics

Innovative insights targeting PSMA, a membrane antigen typically overexpressed in prostate cancer cells, are linked to microRNAs (miRNAs) as a novel tumor-suppressive agent. However, these miRNAs could be entrapped in endocytic compartments and degraded by nucleases, impeding their efficiency as anti-cancer delivery vehicles. Thus, recent research has assessed the antitumor efficacy by developing a construct comprising miR-34a, known for inhibiting PC cell proliferation, a targeting ligand DUPA (2-[3-(1,3-dicarboxy propyl) ureido] pentanedioic acid) specifically for PSMA, and nigericin as endosomal escape agent. The resulting complexes, DUPA-nigericin-miR-34a and DUPA-miR-34a, were evaluated in vitro and in vivo on PSMA-expressing cancer cells. The results show that only DUPA-nigericin-miR-34a has decreased tumor cell proliferation and has delayed tumor growth. These efforts highlight that enrolling miRNAs as anti-cancer agents is a promising endeavor, but non-specificity and even toxicity of therapeutic delivery are the main challenges of this novel approach [102].

To better understand these barriers, recent studies have explored novel ligands coupled to miRNA species and examined antitumor efficacy starting from the premise that prostate cancer stem cells (PCSCs) contribute to the lethality of PC by inducing resistance to therapy. Therefore, hampering PCSC functions could enlarge the current therapeutic arsenal in PCs. Thus, in vitro examination of the antitumor efficacy of the folate-conjugated miR-34a (folate–miR-34a), addressing the folate receptor α (FOLR1), presented interesting results. Hence, it was reported that folate–miR-34a did not inhibit PC growth, in accordance with additional studies that have indicated low FOLR1 expression in PC; thus, the strategy of blocking the folate receptor via ligand-conjugated miR-34 is not appropriate in PC. Surprisingly, not only FOLR1 but also PSMA should be considered as limiting factors for ligand-miR-34a therapeutics, considering the highly heterogenic expression of PSMA in advanced PCs, as described in Section 3.3. These results, however, enlighten advances on the ligand-conjugated (and unconjugated) miR-34a as possible therapeutics for advanced PCa [103].

Challenges in PSMA-based therapies, as described in Section 3.3., could be overcome using molecular tools, like miRNAs, in prostate cancer cases that are difficult to treat.

To summarize the approved diagnostic radionuclides, we underline that, for the initial staging of PC with BR and/or progression, [^18^F]DCFPyL (piflufolastat) and [^68^Ga] Ga-PSMA-11 should be used preponderately in the clinical settings. For therapy and diagnostics of mCRPC, ^177^Lu- is the currently preferred radionuclide for PSMA-targeted therapeutic purposes with its commercially available compounds such as [^177^Lu] Lu-PSMA-617 and [^177^Lu] Lu-PSMA-I&T [104].

## 6. Perspectives and Conclusions

Radiation therapy for prostate cancer has evolved significantly over the past decade, marked by new frontiers in imaging, treatment delivery, and enhanced insights into the pathobiology of this malignancy [105]. The therapeutic arsenal has expanded to include PARP inhibitors and immunotherapy, such as anti-PD1 agents, specifically for castration-resistant prostate cancer that harbors alterations in its DNA repair genes.

Immunotherapy has the potential to synergize with radiotherapy. Radioligand therapy based on ^177^Lu-PSMA-617 can synergize with immune checkpoint inhibitors, leading to enhanced tumor cell killing. The rationale for combining ^177^Lu-PSMA-617 with anti-PD-1/PD-L1 inhibitors reside in a better infiltration of tumors with T CD8+ lymphocytes due to HMGB1 danger protein release that induces immunogenic tumor cell death. In addition, the expression levels for PSMA would be increased [106]. This is a highly explored strategy in several clinical trials; for instance, ^177^Lu-PSMA-617 combined with pembrolizumab (anti-PD-1 molecule) in mCRPC patients prolongs progression-free survival, thus having a favorable impact on the objective response rates [107].

Two recent trials combining ^177^Lu-PSMA-617 and pembrolizumab (NCT03805594, NCT03658447) assessing the safety profile in mCRPC patients have shown better disease control [108] when using a single initial dose of ^177^Lu-PSMA-617 followed by maintenance treatment with pembrolizumab, results that are encouraging for patients with mCRPC [109].

Therapeutic efficacy in PC with bone metastatic disease can be improved by combining chemotherapy with ^177^Lu-PSMA-617. This synergistic approach sensitizes tumor cells to radiation-induced DNA lesions, thus increasing tumor cell death. Furthermore, the combination of ^177^Lu-PSMA-617 and chemotherapy has been approached in some recent clinical trials in mPC [110]. Thus, a phase I/II study evaluating the safety and efficacy combination of ^177^Lu-PSMA-617 with cabazitaxel in mCRPC previously treated but progressed under docetaxel therapy. The primary goal of the trial was to establish the maximum tolerated dose, dose-limiting toxicities, and clinical results when recommending this combination [111]. Another phase II trial will assess the effectiveness of treatment with ^177^Lu-PSMA-617 and docetaxel versus docetaxel alone in patients with newly diagnosed mCRPC. The study’s main endpoint is undetectable levels of PSA at 12 months [112].

The most effective PC treatment depends on cancer’s stage, grade, age, overall health, and genetic patterns. Localized PC is often treated with surgery or radiation, locally advanced cancer, may require more aggressive treatments, e.g., hormone therapy adding to radiation or surgery. Metastatic cancer is typically treated with hormone therapy, chemotherapy, or immunotherapy to manage symptoms and slow progression. Age is another factor that influences the therapeutic option. Age and overall health are important factors, as older patients or those with heart disease and diabetes may not tolerate aggressive treatments well. Younger and healthier patients may be subjected to more aggressive therapies, including surgery or radiation. The genetic pattern can also influence the therapy option. PI3K pathway mutations are the most frequent mutations in PC, followed by the Ras/Raf/MAPK pathway, p53, Rb, and Myc [113]. These mutations can account for the failure of androgen deprivation therapy and guide to other therapeutic approaches, radiotherapy included.

Therapeutic radiopharmaceuticals are a good therapeutic option. Radiation therapy is highly effective for localized prostate cancer and early-stage cancer [114,115]. There are several criteria when choosing from the best three clinically used radionuclides, ^177^Lu, ^225^Ac, and ^223^Ra. Price and availability of the nuclear facility to provide the drug are probably the main criteria for the physician. The actual cost of therapy varies between countries, medical facilities, the number of procedures, the need for hospitalization, and other factors. Table 2 provides an outline of the clinical characteristics of ^177^Lu, ^225^Ac, and ^223^Ra, along with their actual costs and the number of patients treated until now. For various details regarding the domain, there are important review papers. For example, for details concerning ^177^Lu-based therapy, a recently published review focused on its characteristics [116] or its clinical trials [110], or more general clinical nuclear medicine of PC [117], or last, but not least, radiotherapy and radiodiagnostics in PC and the chemistry behind radiolabeling [118].

Advanced technologies, including whole-genome sequencing, transcriptome analysis, epigenetic profiling, and singe-cell RNA sequencing, have the potential to propel prostate cancer into the molecular era, thereby facilitating a deeper understanding of tumor heterogeneity and the characteristics of the tumor microenvironment that may elucidate clinical responses and resistance mechanisms. Monitoring clinical outcomes in PC could benefit from evaluating cell-free DNA, circulating tumor cells, and extracellular vesicles. Furthermore, molecular imaging can enhance precision medicine in prostate cancer by detecting tumor heterogeneity and the progression from primary tumors to metastases, thus identifying novel therapeutic targets [119,120].

Theranostics based on PSMA-targeted radiopharmaceuticals and the combination of treatments, including immunotherapies, are becoming increasingly available for advanced prostate cancer; however, challenges arise as treatment selection becomes more complex [120,121]. A primary clinical challenge in prostate cancer management is the optimal sequencing of therapeutic modalities for patients. Current guidelines often fail to address clinical solutions that consider disparities in radionuclide therapy availability across different regions of the world.

Additionally, prostate cancer’s biological diversity necessitates novel approaches to imaging, monitoring, and comprehensive disease management strategies. The advent of radioligand therapies targeting prostate-specific membrane antigens has fundamentally transformed the therapeutic landscape for prostate cancer patients.

## Figures and Tables

**Table 2:** Clinical characteristics of the main radionuclides used in the theranostics of prostate cancer.

Radionuclide	Treatment Response (% of Tumor Decrease)	PSA Levels (% of Serum PSA Decrease)	Disease Recurrence Rate (% of Decrease)	Average Cost of Treatment in Germany	Nb of Treated Patients
^177^Lu	30–80%	70%	40%	€17,000–€28,000	15,000–30,000
^225^Ac	50–80%	90%	40–60%	€18,000–€29,000	700–900
^223^Ra	30–50%	20–30%	15–30%	€12,000–€15,000	50,000–100,000

## Abbreviations

The following abbreviations are used in this manuscript:

ADCs	Antibody–Drug Conjugates
ADT	Androgen Deprivation Therapy
AR	Androgen Receptor
ARSIs	Androgen Receptor Signaling Inhibitors
BR	Biochemical Recurrence
CRPC	Castration-Resistant Prostate Cancer
cGA	Glucometabolic Activity
CLI	Cerenkov Luminescence Imaging
CT	Computer Tomography
DDR	DNA Damage Repair
DUPA	Dicarboxy Propyl Ureido Pentanedioic Acid
EC	Electron Capture
FAR	Flexible Autoradiography
FDA	Food and Drug Administration
FOLR1	Folate Receptor α
FR	Folate Receptor
GR	Glutamate Receptor
HTRT	Hyperthermia–Radiation Combined Therapy
LET	Linear Energy Transfer
LDR	Low-dose-rate
mCRPC	Metastatic Castration-Resistant Prostate Cancer
miRNAs	Micro RNAs
NAALDase	N-acetylated Alpha-Linked Acidic Dipeptidase
OS	Overall Survival
PARPi	Poly-ADP-ribose Polymerase Inhibitors
PC	Prostate Cancer
PCSCs	Prostate Cancer Stem Cells
PET	Positron Emission Tomography
PFS	Progression-Free Survival
PRMs	Positive Resection Margins
PSA	Prostate-Specific Antigen
PI3K	Phosphoinositide 3-kinases
RFC	Reduced Folate Carrier
RT	Radionuclide Therapy
SBRT	Stereotactic Body Radiotherapy
SGs	Salivary Glands
